# Effect of a mother’s recorded voice on emergence from general anesthesia in pediatric patients: study protocol for a randomized controlled trial

**DOI:** 10.1186/s13063-017-2164-4

**Published:** 2017-09-15

**Authors:** Seok Young Song, Sang Gyu Kwak, Eugene Kim

**Affiliations:** 1 Department of Anesthesiology and Pain Medicine, Daegu Catholic University Medical Center, School of Medicine, Daegu Catholic University, Daegu, Republic of Korea; 2 Department of Medical Statistics, Daegu Catholic University Medical Center, School of Medicine, Daegu Catholic University, Daegu, Republic of Korea

**Keywords:** Emergence agitation, Emergence time, Bispectral index (BIS), General anesthesia, Mother’s voice

## Abstract

**Background:**

Emergence delirium is a behavioral disturbance after general anesthesia in children and may distress both the patients and the primary caregivers, such as parents and medical staff, looking after the patients. Various medical and emotional interventions have been investigated to reduce emergence delirium; however, none are completely effective. This trial intends to assess whether the mother’s recorded voice can reduce this adverse post-anesthesia event and facilitate arousal from general anesthesia.

**Methods/design:**

This is a prospective, double-blind, single-center, parallel-arm, superiority, randomized controlled trial to be conducted in participants aged 2–8 years who are undergoing elective surgery requiring general anesthesia. Participants will be randomly assigned to one of two groups: those who are stimulated to wake up by listening to their mother’s recorded voice (maternal group, *n* = 33) or a stranger’s voice (stranger group, *n* = 33) during anesthetic emergence. The primary outcome is the initial emergence delirium score in the post-anesthesia care unit (PACU). The secondary outcomes are hemodynamic parameters, including heart rate and mean blood pressure, the duration of time between the cessation of anesthetics and a BIS level of 60, 70 and 80, eye-opening or purposeful movement time, extubation time, total consumption of analgesics, PACU stay time, emergence delirium and pain scores during the PACU stay.

**Discussion:**

This is the first randomized controlled trial to investigate the effect of a mother’s recorded voice during emergence on the pediatric emergence profile after general anesthesia. It may provide prophylactic treatment options to decrease emergence delirium and enhance arousal from general anesthesia.

**Trial registration:**

ClicnicalTrials.gov, ID: NCT02955680. Registered on 2 November 2016.

**Electronic supplementary material:**

The online version of this article (doi:10.1186/s13063-017-2164-4) contains supplementary material, which is available to authorized users.

## Background

Emergence delirium (ED) is described as a “mental disturbance during recovery from general anesthesia consisting of hallucinations, delusions and confusion manifested by moaning, restlessness, involuntary physical activity, and thrashing about in bed” [1]. It can cause self-injury, dressing disruptions, surgical dehiscence, and dislodged indwelling catheters and can lead to parents’ and surgeons’ dissatisfaction with the anesthetic. The incidence of ED in pediatric patients after general anesthesia is reported to be between 10 and 80% [2]. Several factors, such as sevoflurane or desflurane anesthesia, younger age, pain, anxiety and the temperament of the child have been suggested to contribute to ED, but the precise etiology remains uncertain [2, 3]. Various studies have tried to reduce emergence delirium after anesthesia [4–6], but none have been completely effective.

Mothers usually spend a large amount of time with their children. Therefore, mothers can be assumed to contribute to their children’s emotional status and neurological development. In a previous study, maternal voice had special importance and enhanced involuntary attention [7]. Moreover, a mother’s voice elicited stronger activations in specific cerebral regions than an unknown voice in another study [8]. A recorded mother’s voice increased the activation of the prefrontal cortex compared to a stranger’s voice [9]. As shown by these studies, a mother’s voice has been shown to have a strong impact on both behavioral and neuronal responses in children.

However, the influence of a familiar voice, especially a mother’s voice, on a child during anesthetic emergence has been scarcely explored previously. Therefore, we will investigate whether maternal voice supports emotional stability and facilitates emergence during the emergence period. We hypothesized that children would show reduced ED and faster emergence when they heard their mothers’ recorded voices compared to a stranger’s recorded voice.

## Methods

### Study design

**Fig. 1:**
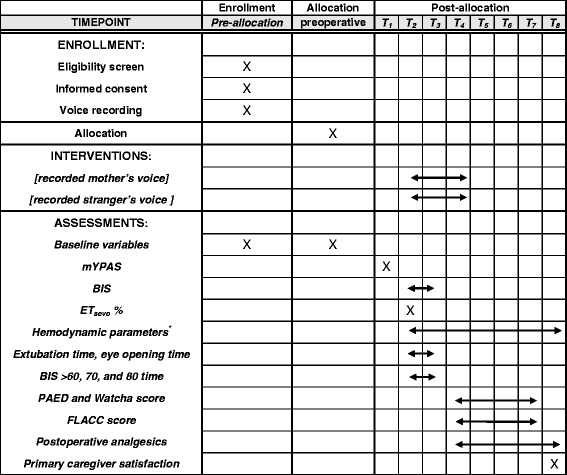
The SPIRIT flow diagram: the schedule of enrollment, interventions and assessments. *including mean blood pressure and heart rate, *T*_*1*_ at preoperative waiting area, *T*_*2*_ cessation of anesthetics, *T*_*3*_ extubation, *T*_*4*_ PACU arrival, *T*_*5*_ 10 min after post-anesthesia care unit (PACU) arrival, *T*_*6*_ 20 min after PACU arrival, *T*_*7*_ 30 min after PACU arrival, *T*_*8*_ PACU discharge, *mYPAS* modified Yale Preoperative Anxiety Scale, *BIS* Bispectral Index, *ET*_*sevo*_ end-tidal sevoflurane concentration, *PAED* Pediatric Assessment of Emergence Delirium, *FLACC* face, legs, activity, cry and consolability

This prospective, single-center, parallel-arm, double-blind, randomized controlled trial was approved by the Institutional Review Board (IRB) of Daegu Catholic University Medical Center (CR-16-139-L) and registered at ClinicalTrials.gov (NCT02955680) before enrollment. This trial is conducted in a tertiary university hospital (Daegu Catholic University Medical Center) in South Korea. We report the protocol of this trial according to the SPIRIT guidelines and the SPIRIT flow chart (Fig. 1 and Additional file 1, respectively). This protocol also follows the CONSORT Statement.

### Participants

Pediatric patients aged 2–8 years with an American Society of Anesthesiologists physical status (ASA PS) of 1 or 2 who are scheduled to undergo ophthalmology or otorhinolaryngology surgery requiring general anesthesia will be enrolled. Potential participants who meet the inclusion criteria will be recruited at outpatient clinics or during preoperative visits before surgery. The exclusion criteria are described as follows:

- ASA PS 3 or 4
- Presence of developmental delays or neurological diseases
- Deafness or hearing impairments
- History of allergies or contraindications to the use of ketamine (increased intracranial pressure, open-globe injury, or a psychiatric or seizure disorder)
- Maternal mutism
- Absence of the mother

### Ethics, consent and permissions

We will obtain written informed consents from all of the legal guardians of the participants via a single investigator (SYS) prior to any study-related procedures.

### Randomization and blinding

**Fig. 2:**
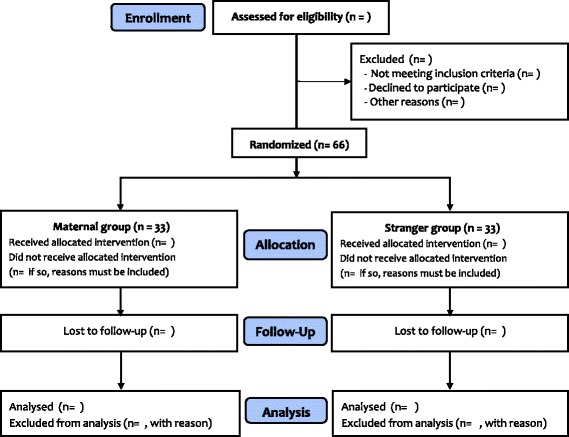
CONSORT flow chart

After recruitment, the participants will be randomized to the maternal group or the stranger group with a 1:1 ratio (Fig. 2) using a computerized online tool (www. Randomization.com). A random sequence will be managed and kept within sealed, opaque envelopes by an assistant who is not involved in this study. When a patient is enrolled in the study, an anesthesia nurse will open a sealed envelope and prepare the mother’s or a stranger’s recorded voice file using a voice recorder according to the group allocation. The voice recorder will be delivered to the operating room after the patient arrives and will be connected to noise-cancelling headphones before surgical draping.

Allocations will be concealed to all investigators and participants except the anesthesia nurse who delivers the voice recorder to the investigator. If serious adverse events that threaten the safety of patients occur (such as death or irreversible injury), we will stop the intervention immediately, break the blinding, and contact the IRB.

### Withdrawal, dropout, and discontinuation

Participants are free to withdraw at any time during the trial at their own or their legal representative’s request. If the continuation of the trial may threaten the participant’s health, a participant can be withdrawn based on the investigators’ judgment. Reasons for withdrawal will be recorded in case report form. All data will be analyzed according to the intention-to-treat principle.

### Confidentiality

Personal information including names, social security numbers or chart numbers will not be collected. Only the study code will be collected and managed separately. The collected data will remain confidential until the investigators analyze the data. The final dataset will be managed by the chief investigator (EK). After the completion of the study, the collected data will be encrypted and stored for 3 years and then discarded.

### Intervention

At the preoperative waiting room, patients are premedicated with atropine (0.02 mg/kg) and ketamine (1 mg/kg) administered intravenously in the parental presence. After a patient enters the operating room, standard monitoring (electrocardiography, noninvasive arterial blood pressure, and pulse oximetry) and the Bispectral Index (BIS; VISTATM monitoring system; Aspect Medical Systems Inc., Norwood, MA, USA) will be applied. Anesthesia will be induced using fentanyl (1 mcg/kg) and 5–6% of sevoflurane via a scented facial mask. Rocuronium 0.6–0.8 mg/kg will be given to facilitate intubation. Anesthesia will be maintained with 1.5–2.0 MAC of sevoflurane and 50% oxygen, with a BIS target range of 40–60. At the end of the operation, pyridostigmine and glycopyrrolate will be administered to antagonize neuromuscular blockade. All patients will be given prophylactic antiemetics with dexamethasone 0.1 mg/kg and ondansetron 0.1 mg/kg.

According to the pre-assigned allocation, the patients will be stimulated to wake up by a repeated recording of the mother’s voice (maternal group) or a stranger’s voice (stranger group) at the end of the operation. The voice recording will be performed before the operation. In a calm environment, the mother or a woman who does not know the child is asked to speak the following sentences in her usual tone of voice:

“OO (first name of child), wake up. Let’s go home with mommy. OO, wake up. Open your eyes. Take a deep breath.”

Only light patting on the shoulder and the recorded voice will be allowed to stimulate the patients. The recorded voice will be delivered to the patient repeatedly via noise cancelling headphones until extubation. An investigator who is blinded to the assigned intervention (EK) will check the BIS value and the end-tidal sevoflurane concentration (ET_sevo_ %) at the end of surgery, the extubation time, the eye opening or purposeful movement time and the time to BIS values of > 60, 70, and 80 during the emergence period. The BIS value will be included if the electromyography score is < 50% and the Signal Quality Index is > 75%. After gentle suctioning of oral secretions from the oropharynx, extubation will be carefully performed when the participants are able to achieve spontaneous breathing and obey verbal commands. Voice stimulation via headphones will be maintained until the patients arrive at the post-anesthesia care unit (PACU).

### Measurements

The primary outcome measurement is the initial PAED score in the PACU. The secondary outcomes are hemodynamic parameters (heart rate, mean blood pressure), the duration of time from the cessation of anesthetics to a BIS score of 60/70 and 80 (BIS > –60/70/80 time), the eye-opening or purposeful movement time, the extubation time, the total consumption of analgesics, PACU stay time, and PAED, Watcha, and FLACC scores in the PACU.

The Modified Yale Preoperative Anxiety Scale (m-YPAS) will be used when the patients arrive at the preoperative waiting room with their parents before premedication [10, 11]. At the end of surgery, all anesthetics will be stopped immediately and the BIS value and the end-tidal sevoflurane concentration (ET_sevo_ %) are recorded. Hemodynamic parameters, including heart rate (HR) and mean blood pressure (MBP), will be checked at five time points; at the time of the cessation of anesthetics (baseline, t_0_), time to reach a BIS value of 60 (t_1_), time of extubation (t_2_), time of PACU arrival (t_3_), and time of PACU discharge (t_4_). BIS > 60, 70, and 80 times, defined as the time intervals from the cessation of anesthetics until the BIS reached values of 60, 70, and 80, respectively, will be measured. Eye-opening or purposeful movement time is defined as the interval from the cessation of anesthetics to-eye opening or purposeful movement of the patient. Extubation time, defined as the interval from the cessation of anesthetics until extubation, will also be measured.

Upon arrival to the PACU, a blinded investigator will measure emergence delirium and pain scores at every 10 min for the first 30 min. Emergence delirium will be measured by two methods, the Pediatric Assessment of Emergence Delirium (PAED) and the Watcha scales. The PAED scale has five categories (eye contact, actions, awareness, restlessness, and consolability) scored from 0 to 4 and summed to obtain a total score with a range of 0–20 [12]. The degree of emergence delirium increased directly with the total scores. The Watcha scale is a four-point scale as follows [13]: 1, calm; 2, crying but can be consoled; 3, crying and cannot be consoled; 4, agitated and thrashing around. A total PAED score > 12 or a Watcha score > 2 at any time is considered post-anesthetic ED. Pain will be measured by Face, Legs, Activity, Cry and Consolability (FLACC) scores ranging from 0 to 15 [14]. In patients with a total PAED score > 12, a Watcha score > 2 or a FLACC score > 4 in the PACU, fentanyl 0.5 mcg/kg will be administered as a rescue medication and repeated if the agitation does not subside. Adverse events, such as postoperative nausea and vomiting, excessive secretions, laryngospasm, or desaturation and the total consumption of analgesics, will also be measured. The patients will be discharged when they are calm and meet a modified Aldrete score ≥ 9 [15], and the duration of the PACU stay will be recorded as the PACU stay time. The subjective satisfaction of the primary care giver such as a parent or the PACU nurse will be measured just before PACU discharge on a five-point scale as follows: 1, very disappointed; 2, disappointed; 3, so-so; 4, satisfactory; 5, very pleased.

### Sample size

The primary outcome is the initial PAED score. In our pilot study, the mean difference of the PAED score between the stranger group and the maternal group was 1.6 with a standard deviation (SD) of 2.19. To attain an alpha value of 0.05 and a test power of 0.8, 30 patients are needed in each group using the following formula [16]. We plan to enroll a total of 66 patients to allow for a 10% dropout rate:

$$ n\kern0.5em =\kern0.5em \frac{2{\left({z}_{a/2}+{z}_{\beta}\right)}^2\kern0.5em \cdotp \kern0.5em {\sigma}^2}{{\left({\mu}_c-{\mu}_t\right)}^2} $$

To compensate for the possibility of an over-estimated effect size of the pilot study, we will perform an interim analysis at 50% enrollment. If the sample size has significantly lower power than our expectation, we will recruit more patients. Otherwise, we will continue the study according to our initial sample size calculation.

### Statistical analysis

An intention-to-treat analysis will be performed and missing data will be input with the last-observation-carried-forward method. Continuous variables will be presented as the mean and the SD or the median and the interquartile range (IQR) according to the normality assumption using the Kolmogorov-Smirnov test. Categorical variables will be presented as numbers of patients and proportions.

The primary outcome (the initial PAED score) will be compared with an independent *t* test or a Mann-Whitney *U* test. For secondary outcomes, continuous variables will be compared with an independent *t* test or the Mann-Whitney *U* test and categorical variables will be compared with Pearson’s chi-squared test or Fisher’s exact test. All tests are two-sided and *P* < 0.05 is considered statistically significant. A statistician (SGK) who is not involved in the data collection will conduct all statistical analyses with SPSS software (version 21.0; SPSS Inc., IBM, Chicago, IL, USA).

## Discussion

The effect of a mother’s voice elicited positive physiologic and behavioral responses in other clinical territories [17–19], but this has been scarcely reported in perioperative settings. In a study by Kim and colleagues [20], a mother’s recorded voice reduced preoperative anxiety and emergence agitation during cardiac catheterization using ketamine sedation. However, the quality of mothers’ voices was not standardized and the control group did not receive any voice stimuli. In contrast, we had the mothers read the same dialogue in a calm environment in all study populations and compared the mothers’ voices with the stranger’s voice. Additionally, we measured the BIS to quantify the sedation level and the effects of a mother’s voice on the emergence of her child compared with a stranger’s voice. Other major differences are the level of anesthesia and the surgical stimuli. Our study involves investigation under general anesthesia using both ketamine and sevoflurane inhalation, but Kim’s study was conducted under modest sedation using ketamine only [20]. Sevoflurane or desflurane anesthesia is believed to contribute to a higher frequency of emergence delirium [21].

In this study, patients are premedicated with ketamine before entry to the operating room. Various agents have been shown to be beneficial effect on prevention of ED [3–6, 22–24]. Among the numerous drugs, a new selective α_2_-agonist, dexmedetomidine has recently been spotlighted as an effective prophylactic and treatment agent for ED [2, 6, 22]. Despite the ongoing encouragement from the previous findings, however, there have been no officially approved indications for its use in the pediatric population. Meanwhile, ketamine has been safely used in pediatric anesthesia for over five decades. It effectively decreased the incidence of emergence delirium in previous studies [23, 24]. Moreover, it was equally effective as dexmedetomidine in a study after strabismus surgery with sevoflurane [25]. Taken all together, we thought that ketamine would be suitable for our settings.

To our knowledge, this is the first randomized controlled trial to investigate the advantage of a mother’s recorded voice during the emergence period on the pediatric emergence profile after general anesthesia. If the study is successful, this research will establish a new prophylactic option to decrease emergence delirium and enhance the wake up after general anesthesia.

### Trial status

Patient recruitment is still ongoing since November 2016.

## Additional file

Additional file 1: SPIRIT Checklist. (DOC 123 kb)

## Abbreviations

BIS: Bispectral Index
FLACC: Face, Legs, Activity, Cry and Consolability
m-YPAS: Modified Yale Preoperative Anxiety Scale
PACU: Post-anesthesia care unit
PAED: Pediatric Assessment of Emergence Delirium
